# Effects of training of shadowing and reading aloud of second language on working memory and neural systems

**DOI:** 10.1007/s11682-020-00324-4

**Published:** 2020-07-23

**Authors:** Hikaru Takeuchi, Tsukasa Maruyama, Yasuyuki Taki, Kosuke Motoki, Hyeonjeong Jeong, Yuka Kotozaki, Takamitsu Shinada, Seishu Nakagawa, Rui Nouchi, Kunio Iizuka, Ryoichi Yokoyama, Yuki Yamamoto, Sugiko Hanawa, Tsuyoshi Araki, Kohei Sakaki, Yukako Sasaki, Daniele Magistro, Ryuta Kawashima

**Affiliations:** 1 Division of Developmental Cognitive Neuroscience, Institute of Development, Aging and Cancer, Tohoku University, 4 − 1 Seiryo-cho, Aoba-ku, Sendai, 980−8575 Japan; 2 Faculty of Medicine, Tohoku University, Sendai, Japan; 3 Department of Community Medical Supports, Tohoku Medical Megabank Organization, Division of Medical Neuroimaging Analysis, Tohoku University, Sendai, Japan; 4 Department of Nuclear Medicine and Radiology, Institute of Development, Aging and Cancer, Tohoku University, Sendai, Japan; 5 Graduate School of International Cultural Studies, Tohoku University, Sendai, Japan; 6 Japan Society for the Promotion of Science, Tokyo, Japan; 7 Division of Clinical research, Medical-Industry Translational Research Center, Fukushima Medical University School of Medicine, Fukushima, Japan; 8 Division of Psychiatry, Tohoku Medical and Pharmaceutical University, Sendai, Japan; 9 Human and Social Response Research Division, International Research Institute of Disaster Science, Tohoku University, Sendai, Japan; 10 Smart Ageing International Research Center, Institute of Development, Aging and Cancer, Tohoku University, Sendai, Japan; 11 Department of Psychiatry, Tohoku University Graduate School of Medicine, Sendai, Japan; 12 School of Medicine, Kobe University, Kobe, Japan; 13 Advanced Brain Science, Institute of Development, Aging and Cancer, Tohoku University, Sendai, Japan

**Keywords:** Shadowing, Reading aloud, Training, Brain structure, Working memory, Brain activity

## Abstract

Shadowing and reading aloud both involve multiple complex cognitive processes, and both are considered effective methods for second-language learning. The working memory system, particularly the phonological loop, has been suggested to be involved in shadowing and reading aloud. The purpose of this study was to investigate the effects of a 4-week intensive adaptive training including shadowing and reading aloud of second language on working-memory capacity, regional gray matter volume (rGMV), and functional activation related to the n-back working-memory task in young adults. The results showed that compared with the training groups without speaking (listening to compressed speech and active control involving the second language), the training groups with speaking (shadowing and reading aloud) showed a tendency for greater test-retest increases in digit-span scores, and significantly greater test-retest decreases in N-back task reaction time (increase in working memory performance). Imaging analyses revealed compared with the active control group, shadowing group exhibited decreases in rGMV and brain activity during the working memory task (2-back task), in the left cerebellum and reading group exhibited decreases in them in the right anterior insula. These regions are parts of the phonological loop, suggesting the presence of training-induced neural plasticity in these neurocognitive mechanisms.

**Electronic supplementary material:**

The online version of this article (10.1007/s11682-020-00324-4) contains supplementary material, which is available to authorized users.

## Introduction

Shadowing is defined as a listening act or task in which the learner tracks the heard speech and repeats it as exactly as possible while listening attentively to the incoming contextual information. Shadowing is a complex act involving auditory input, speech output, repetition, and divided attention. Particularly, the involvement of the working-memory system, especially that of the phonological loop, in shadowing has been widely implicated (Gyselinck et al. 2002; Hamada 2015). The phonological loop is assumed to hold verbal and acoustic information using a temporary storage and an articulatory rehearsal system (A. D. Baddeley 2017).

As for the neural bases of shadowing and the relevant cognitive mechanisms involved, the phonological loop which is suggested to be important for act of shadowing, was suggested to include the perisylvian areas, inferior parietal lobule, and the cerebellum (Osaka and Nishizaki 2000; A. Baddeley 2003; Aboitiz et al. 2010). Previous neuroimaging studies showed that widespread perisylvian areas and the cerebellum were activated during the act of shadowing (Peschke et al. 2009; Tommola et al. 2000).

Training of shadowing has gained attention as a tool for learning a second language. On the other hand, shadowing is widely accepted as one of the means to improve second-language learners’ listening skills during interpreter training (Kurz 1992; Hamada 2012) and foreign-language learning (Hamada 2016). In addition, interpreters, who are engaged in shadowing while being trained as interpreters show increased working-memory capacity (Liu et al. 2004; Signorelli et al. 2012). Furthermore, previous intervention studies showed that shadowing training led to improved listening (listening comprehension skills and phoneme perception) compared with dictation training in low-level second-language learners, but not in high level-learners (Tamai 2002; Hamada 2016).

Previous studies have not utilized the intensive adaptive training methods on shadowing training. Intensive adaptive training involves the adjustment of difficulty of the training tasks and concentrated training schedules. Here, adaptive means training is tailored close to the individual’s capacity by adjusting the difficulty based on the performance. In addition, intensive training means training is conducted with a heavy schedule of practice trials. Intensive adaptive training has been shown to enhance training effects for both perceptual and motor learning, and to induce cortical plasticity (Buonomano and Merzenich 1998; Tallal et al. 1996). For training of working memory, several attempts to improve working memory, including learning through the use of effective strategies, generally failed to improve performance on tasks other than the tasks that the individuals were trained for (Kristofferson 1972; Phillips and Nettelbeck 1984). However, intensive adaptive training of working memory has been shown to induce improved performance in areas other than the trained and closely-related tasks (Klingberg et al. 2002; Au et al. 2016). This method has been applied to training for higher order cognitive functions (Hikaru Takeuchi et al. 2014). In addition, previous studies have not investigated the effects of shadowing training on working-memory capacity, brain structures, and neural systems related to working memory.

Another method shown to improve second-language skills is reading aloud (Gibson 2008). Reading aloud is a complex act involving visual input, speech output, and divided attention. Considering the cognitive model, the commonalities of reading aloud and shadowing involve divided attention and fluent speech output. However, the difference is that reading aloud requires automatic visual phonetic coding and sentence recognition whereas shadowing requires automatic recognition of auditory speech. Similar to shadowing, neural systems of reading aloud include the perisylvian areas and overlap with the neural system of phonological loop of working memory (Graves et al. 2010). However, unlike shadowing, reading aloud involves the visual cortex and left angular gyrus corresponding to the visual input of texts (Miura et al. 2003). Combined cognitive training, including reading aloud of L1 and other training tasks, is known to enhance some cognitive functions, such as working memory (Nouchi et al. 2012; Nouchi et al. 2013). However, the effects of training using reading aloud alone on working-memory capacity, as well as on brain structures and neural systems related to working memory, are unclear.

The purpose of this study was to investigate the effects of intensive adaptive training of shadowing and reading aloud of second language on working-memory capacity, brain structures, and neural systems related to working memory. Considering the relevance of shadowing and reading aloud to subsystems of both working memory and second-language learning, it is important to investigate the extent of cognitive and neural plasticity caused by these two methods.

Based on the demonstrations of (a) the associations between shadowing and working memory, (b) the associations between reading aloud and working memory, and (c) overlapping neural systems of the phonological loop supporting both reading aloud and shadowing in previous studies as described above, we hypothesized that training in intensive adaptive shadowing and reading aloud second language would lead to improvements in working-memory capacity, functional activation, and gray matter structural changes in the neural systems of the phonological loop, namely in the perisylvian areas and the cerebellum.

We utilized two representative methods for studying neural plasticities for investigating use-dependent regions. One is voxel-based morphometry (VBM), which is a powerful, sensitive, and widely used method that can evaluate structural plasticities over the entire brain (Draganski et al. 2004). The other is a functional magnetic resonance imaging (fMRI) scan of N-back working memory task, which is the representative task for tapping brain activity during working memory (Owen et al. 2005). By utilizing these two approaches, we aimed to reveal use-dependent neural plasticity in a comprehensive and sensitive manner.

## Methods

Subjects. The study cohort included 119 healthy, right-handed undergraduate or graduate students (67 men and 52 women; mean age, 20.6 ± 1.58 years) with normal vision and no history of neurological or psychiatric illness, as assessed with a routine questionnaire in which each subject answered questions about a history of certain illnesses. Handedness was evaluated using the Edinburgh Handedness Inventory (Oldfield 1971). In this experiment, the inclusion criteria included English skills (EIKEN, an English proficiency assessment that is widely used in Japan), Grade Pre-2 (mid-high-school level or greater), or a Test of English for International Communication (TOEIC) score of >400). The criteria can be considered to be intermediate level of ability used by the Ministry of Education, Culture, Sports, Science, and Technology (MEXT) as a recommended benchmark for high-school graduates in Japan. Written informed consent was obtained from each subject, and the study protocol was approved by the Ethics Committee of Tohoku University and performed in accordance with the tenets of the Declaration of Helsinki (1991).

The data from this experiment was used in another study that compared the training effects of time-compressed speech in second language (TCSSL) with the active-control group (Maruyama et al. 2018). In this previous study (Maruyama et al. 2018), the effects of listening to time-compressed speech were examined by comparison with the active control group, whereas in the present study, the effects of shadowing and reading aloud, both of which involved speaking (unlike just listening to compressed speech), were investigated. By utilizing the data on the training effects of TCSSL, we can exclude the possibility that the effects seen in the shadowing group in this study were caused by listening to the faster speed of the second language.

Participants were randomly assigned to one of the four groups: the training with time-compressed speech of a second-language group, a shadowing training group, a reading-aloud training group, or an active-control group. Four interventions were performed in parallel, and this study design does not involve a crossover design and participants of each group were exposed to only one type of training and with no interventions subsequently. None of the participants in the active-control group were notified that they belonged to the “control” group until after the post-magnetic resonance imaging (MRI) and psychological examinations. The training for shadowing consisted of 30 participants (20 men and 10 women), with a mean age of 20.3 ± 1.4 years. Training for reading aloud consisted of 29 participants (19 men and 10 women), with a mean age of 20.7 ± 1.7 years. The training with time-compressed speech of a second-language group consisted of 30 participants (12 men and 18 women) with a mean age of 20.7 ± 1.5 years. The active-control group consisted of 30 participants (16 men and 14 women) with a mean age 20.8 ± 1.7 years). Analyses of variance (ANOVA) revealed no significant differences in the demographics among the groups (*P* > 0.05), such as age (*P* = 0.587, F = 0.647); score of the Raven’s Advanced Progressive Matrix (Raven 1998), which measures cognitive ability, which is central to general intelligence (Snow 1989) (*P* = 0.226, F = 1.472); or scores of the English listening (*P* = 0.905, F = 0.188) and reading tests (*P* = 0.946, F = 0.123) (described below). Fisher’s exact test revealed no significant differences in sex ratio among the four groups (*P* = 0.135). One participant from the training with TCSSL group, one participant in the active-control group, and one participant in the reading-aloud training group withdrew from the study after the pre-MRI and psychological experiments, and did not participate in the post-MRI and psychological experiments. Another participant in the shadowing training group who could not submit the training data (for the provision of the training data, see below) was also excluded from the study. Thus, 29 subjects in the training with TCSSL group, 29 subjects in the active-control group, 28 subjects in the reading-aloud training group, and 28 subjects in the shadowing training group successfully completed the study. Parts of the descriptions in this paragraph were reproduced from our previous study focusing on TCSSL (Maruyama et al. 2018).

### Procedure

Subjects underwent training for approximately 4 weeks (27 days) for 30–60 min each day in most cases. The total training time was not pre-established; however, it was determined by the amount of tasks the subjects had to complete and thus was dependent on the level of the task and the time between trials in the three groups (shadowing, TCSSL, and active control). In these three groups, the subjects used the auditory English stimuli files provided to them on their personal computers, and they were instructed to perform the task at home every day. The tasks for subjects in these three groups included listening to each English auditory stimulus and recording their responses to the tasks in their computer files. In the reading-aloud group, subjects were instructed to read aloud the given text as fast as possible while doing the instructed task, and to record their voice for reading aloud and their responses to the task, in their computer files. Thus, training duration depended on the speed of the individual subject’s reading ability. The subjects were allowed to miss a session because of computer problems, illness, or other reasons and could complete the task more than once per day. The details of the task are described below. Almost every day, the subjects uploaded the logs to the shared folders for compliance verification. The experimenter provided training feedback to the subjects as necessary (such as contacting the subjects when there was no upload for a while). MRI and psychological examinations were performed immediately before and after the 4-week training course. More specifically, pre-training MRI scans and psychological tests were performed on day 1, training was provided from day 2 to day 28, and post-training MRI scans and psychological tests were performed on day 29. For the actual number of training days completed by the subjects, see the training data subsection in the Methods section. Note that the subjects were students who were exposed to English in classes or other places, at least to some extent. However, this was a randomized controlled study and like any other numerous factors that can affect outcome measures, such points are not supposed to affect group differences of those outcome measures. Some portions of the descriptions in this subsection were reproduced from our previous study comparing the effects of TCSSL with the active-control group (Maruyama et al. 2018).

### Training tasks

Some auditory English stimuli files (for the shadowing, TCSSL, and active control) and English texts (for the reading aloud) were provided to each participant based on their initial English proficiency level. For details, see our Supplemental Methods.

- In the training with TCSSL group, the playing speed was modulated according to the performance of the task, as described in our previous study (Maruyama et al. 2018). In this group, six auditory stimulus files were provided. Subjects were asked to perform the confirmation task while listening to the files. Based on confirmation task performance while listening to each auditory file, the speed of the next auditory file was modulated. The details are presented in the Supplemental Methods.
- In the shadowing training group, 4 auditory stimulus files were given to subjects each day. The confirmation tasks were similar to the training tasks of TCSSL. However, in the shadowing group, in addition to the procedure of the training tasks of TCSSL, subjects were asked to perform shadowing (reading aloud the sentences they heard from the auditory English stimuli). And based on the performance of confirmation tasks, and subjective rating of how much subjects could do shadowing, while listening to each auditory file, the speed of the next auditory file was modulated. The details are presented in the Supplemental Methods.
- In the reading-aloud group, subjects were asked to read aloud the given English text as fast as possible. The given text was the same as those of the auditory stimuli for the other three groups. We chose 10 English target sentences (such as “Smartphones are ruining the concert-going experience”) from each stimulus and translated each into Japanese. The subjects were given the text files comprising 10 of these translated Japanese target sentences and assigned question numbers 1–10, for which the subjects were instructed to identify the English target sentences that corresponded to the given 10 Japanese target sentences, while they read aloud the given English text, They were then asked to record their voice saying “Yes” when they found the given Japanese target sentence. If the subjects were unable to identify the given target sentence, they were asked to ignore the target sentence and proceed to the next given target sentence. There were 10 target sentences per English file, and each day the subjects in the reading aloud training group had to read aloud three English texts and were instructed to complete the associated tasks.
  Subjects were asked to read aloud as fast as possible and to record their voices while maintaining the task performance of the associated task level of at least 50%. When it became difficult to achieve the required performance, they were asked to slow their reading speed so that they could achieve the performance level of 50% in the next trial. The rationale for this procedure is the same as for other training tasks and was designed to keep the speed as fast (and demanding) as possible while maintaining comprehension of the second language. Therefore, subjects were not allowed to read aloud articles so fast that they could not understand what they were reading.
- In the control group, the pitch was modulated according to the performance of the task such that subjects had to listen to the English auditory files as high in pitch as possible while maintaining their task performance. Three auditory files were given to subjects each day. The confirmation task was same as those of the TCSSL group. Based on confirmation task performance while listening to each auditory file, the speed of the next auditory file was modulated. The details are presented in the Supplemental Methods.
  The rationale for this method was to give subjects of the control group the adaptive training tasks (the difficulty was modulated based on the performance) using the second language auditory stimuli. However, in this case task, difficulty is modulated at the perceptual level rather than at the level of comprehension (i.e., difficulty in understanding the auditory second language stimuli when presented quickly).

Psychological outcome measures. For the evaluation of pre-training and post-training, a battery of neuropsychological tests and questionnaires was administered. The specific battery of cognitive tests in this study was chosen for this study for specific and unspecific theoretical and practical reasons. The theoretical and study-specific reasons included the hypotheses described in this study. The practical reasons included (a) availability in Japan, (b) ease to administer to several participants at once, and (c) use of common tests across the different intervention studies (e.g., H. Takeuchi et al. 2013a and b) as much as possible to allow possible comparisons across studies (for reference) as well as for experimenters to recognize the pitfalls and characteristics of the cognitive tests before the study. As described in our previous study (Hikaru Takeuchi et al. 2014a and b), other than the neuropsychological tests for the hypothesis, we have administered a wide range of cognitive tests to investigate the effects of interventions in an exploratory nature. This battery included the following contents: [A] Raven’s Advanced Progressive Matrices (Raven 1998), which is a nonverbal reasoning task. [B] A (computerized) digit-span task, a verbal WM task. Subjects were asked to view a progressively increasing number of random digits visually presented one-digit per second on a computer screen. They were then asked to repeat the sequence by pressing numbered buttons on the screen in the presented order (digit-span forward) or in the reverse order (digit-span backward), starting from two digits. Three sequences were given at each level, until the participants responded incorrectly to all three sequences, at which point the task was ended. The score of each test is equal to the sum of the number of digits correctly repeated in the digit span forward and digit span backward tasks. The descriptions of this task were reproduced from our previous study (for the detail of this task, seeH. Takeuchi et al. 2011c).

[C] The Stroop task (Hakoda’s version) (Hakoda and Sasaki 1990; H. Takeuchi et al. 2012), which measures response inhibition and impulsivity. Hakoda’s version is a matching-type Stroop task that requires the subjects to check whether the chosen answers are correct, unlike the traditional oral naming Stroop task. The test consists of two control tasks (Word-Color task and Color-Word task): a Stroop task and a reverse-Stroop task. In this study, we used the Word-Color and Color-Word tasks as measures of simple processing speed and the Stroop and reverse-Stroop tasks as measures of inhibition. For each task, the subjects were instructed to complete as many of these exercises as possible in 1 min. For further details, see our previous work (H. Takeuchi et al. 2012). [D] Arithmetic tasks, similar to those designed by Grabner et al. (2007), which measured multiplication performance and consisted of two forms of one-digit times one-digit multiplication problems (a simple arithmetic task with numbers between 2 and 9) and two forms of two-digit times two-digit multiplication problems (a complex arithmetic task with numbers between 11 and 19). The two forms of each task were the same, but the numbers used in the problems were ordered differently. Each form of the simple and complex arithmetic tasks had to be completed in 30 s and 60 s, respectively. [E] The SA creativity test (Society_For_Creative_Minds 1969), which measures creativity through divergent thinking, involves three types of tasks (generate unique ways of using typical objects, imagine desirable functions for ordinary objects, and imagine the consequences of unimaginable things happening). The SA test scores the four dimensions of the creative process (fluency, originality, elaboration, and flexibility) (H. Takeuchi et al. 2010), and the sum of the graded scores of the four dimensions was used for the analysis. [F] A Japanese reading comprehension task that was developed by Kondo et al. (2003), for which the subjects were instructed to read articles and then answer four questions about the contents of the article by choosing the correct response from five possible answers (fore more details, see Hikaru Takeuchi et al. 2015). [G] A listening-span task, for which simple sentences that state facts are presented successively, and the subjects are instructed to judge whether the sentences are correct and to remember the words that were at the start of each sentence. Each level has three trials, and the levels increase from 2 to 7. The number of trials in which the subjects remembered all the words in the correct order and judged the correctness of all sentences were recorded and used for analyses. [H] the TOEIC exam (Nakamura et al. 2008) to test English reading and listening. We used the listening and reading tests of this practice exam. The listening test consists of four parts that the subjects can hear only once, and the subjects respond to a total of 100 questions. The entire listening test lasts for approximately 45 min. The subjects hear the dialog of the question and are then instructed to choose the correct answer about the dialog from several options. The reading test, which takes approximately 60 min to complete, consists of three parts, for which the subjects read a short or long text and then choose an appropriate answer for a total of 100 questions.

Several questionnaires designed to assess the traits or states of the subjects were collected but are not described in this study. Other than the self-reported questionnaires, all neuropsychological assessments were performed by postgraduate and undergraduate students who were blinded to the group membership of the participants. The descriptions in this subsection were reproduced from our previous study (Maruyama et al. 2018).

#### fMRI tasks

To map training-induced changes in brain activity related to simple cognitive processes and WM, functional magnetic resonance imaging (fMRI) was used. The n-back task is a typical task for fMRI studies with conditions of 0-back (simple cognitive processes) and 2-back (working memory). The n-back task was performed during an fMRI scanning, as described in our previous study (H. Takeuchi et al. 2011a and 2011b; H. Takeuchi et al. 2014a and b). We used a simple block design and the n-back WM task (Callicott et al. 1999) to tap brain activities during the WM task. There were two conditions (0- and 2-back). The subjects were instructed to recall stimuli [visually presented four types of Japanese letters of vowels) seen “n” times previously. In the 0-back task, subjects were instructed to determine whether each presented letter was the same as the target stimulus. For details see Supplemental Methods.

#### Image acquisition

MRI data acquisition was performed using a 3 T Philips Achieva scanner (Philips Healthcare, Andover, MA, USA). Forty-two transaxial gradient-echo images (echo time = 30 ms, flip angle = 90°, slice thickness = 3 mm, FOV = 192 mm, matrix = 64 × 64) covering the entire brain were acquired at a repetition time of 2.5 s, using an echo planar sequence. For the n-back session, 174 functional volumes were obtained. Diffusion-weighted data were acquired using a spin-echo planar imaging (EPI) sequence with the following settings: TR = 10,293 ms, TE = 55 ms, FOV = 22.4 cm, 2 × 2 × 2 mm^3^ voxels, 60 slices, SENSE reduction factor = 2, and number of acquisitions = 1. The diffusion weighting was isotropically distributed along 32 directions (*b* value = 1000 s/mm^*2*^). Additionally, three images with no diffusion weighting (*b* value = 0 s/mm^2^) (b = 0 images) were acquired using a spin-echo EPI sequence (TR = 10,293 ms, TE = 55 ms, FOV = 22.4 cm, 2 × 2 × 2 mm^3^ voxels, and 60 slices). From the collected images, fractional anisotropy and mean diffusivity maps were calculated. These maps were used for the normalization of fMRI images in this study.

High-resolution T1-weighted structural images (240 × 240 matrix, TR = 6.5 ms, TE = 3 ms, FOV = 24 cm, slices = 162, slice thickness = 1.0 mm) were collected using a magnetization-prepared rapid gradient-echo sequence. All subjects who participated in this study also participated in other studies or projects, and MRI scans not described in this study were performed together with those that are described.

#### Pre-processing and data analysis for functional activation data

Pre-processing and data analysis of functional activation data were performed using statistical Parametric Mapping software (SPM8; Wellcome Department of Cognitive Neurology, London, UK) implemented in Matlab (Mathworks Inc., Natick, MA, USA). Prior to the analysis, the BOLD images from the pre-training scan and the BOLD images from the post-training scan were re-aligned and resliced to the mean image of the BOLD images from the pre-training scan. They were then corrected for slice timing, coregistered, and resliced to voxel space of images of diffusion tensor imaging. Subsequently, using a previously validated two-step new segmentation algorithm of diffusion images and the previously validated diffeomorphic anatomical registration through exponentiated lie algebra (DARTEL)-based registration process (Hikaru Takeuchi et al. 2013a and b), all images were normalized. The voxel size of normalized BOLD images was 3 × 3 × 3 mm^3^ and taken to the second-level analyses of functional activities. The rationale for using DTI images in the normalization procedure of BOLD images is provided in Supplemental Methods.

The following procedures for functional activation data analysis were mostly reproduced from our previous study using the exact same methods (H. Takeuchi et al. 2011a). A design matrix was fitted to each participant with one regressor for each task condition (0-, 2-back in the n-back task) using the standard hemodynamic response function. Further, the motion parameters estimated by the realignment step in SPM were added as 6 regressors (3 for translation and 3 for rotation) to represent a linear model for the residual effects of head motion after re-alignment (Karl J Friston et al. 1996a and b). The design matrix weighted each raw image according to its overall variability to further reduce the impact of movement artifacts (Diedrichsen and Shadmehr 2005). The design matrix was fit to the data for each participant individually. After estimation, beta images were smoothed (8 mm full-width half-maximum) and taken to the second level or subjected to a random effect analysis. We removed low-frequency fluctuations with a high-pass filter using a cutoff value of 128 s. The individual-level statistical analyses were performed using a general linear model. An FWHM of 8 mm is widely used in the field for smoothing and was used as a default in our previous fMRI analyses (Takeuchi et al., 2011a, b; Takeuchi et al., 2014a and b) if there were no reasons to apply other values.

In the individual analysis we focused on the activation change and compared activation related to the conditions (0−/2-back versus rest) before and after the intervention period. The resulting maps for each participant representing changes between the pre-measures and post-measures in brain activity during the corresponding conditions, as well as the brain activity during the corresponding conditions in the pre-measures, were then forwarded to a group analysis.

Due to failures in capturing good-quality imaging data and to procedural mistakes during obtaining of the behavioral data, the group-level analyses were performed using data from 28 subjects from the active-control group, 25 subjects from the TCSSL group, 26 subjects from the reading-aloud group, and 27 subjects from the shadowing group.

### Pre-processing of structural data

Pre-processing of imaging data was performed using SPM12 implemented in Matlab. Using the Pairwise Longitudinal Registration toolbox (Ashburner and Ridgway 2012), the midpoint average template images were generated for each participant as well as maps of differences between the Jacobian determinants and of pre-intervention and post-intervention T1-weighted structural images using default parameter settings. The midpoint average image of each subject was then segmented, and the Thorough Clean option was used to remove any odd voxels. Affine regularization was performed in accordance with the East Asian brain with a sampling distance (the approximate distance between the sampled points when estimating the model parameters) of 1 mm. Default parameter settings were used for other parameters in the segmentation process. Then, by multiplying maps of differences between the Jacobian determinants of pre-intervention and post-intervention images and gray matter segments of the midpoint average image of each subject, images of gray matter volume change were generated. Then, diffeomorphic anatomical registration was performed using DARTEL processes. Here we used imported DARTEL images of gray matter and white matter for the processes created above. The DARTEL template was created from the entire set of subjects in this experiment, and the default parameter settings were used for the DARTEL process. Then, using the parameters generated for each participant, the images of gray matter change were normalized and smoothed by convolving each with an isotropic Gaussian kernel of 8 mm FWHM.

#### Statistical thresholds for group-level analysis of imaging and behavioral data

The behavioral data were analyzed using the Statistical Package for the Social Sciences (SPSS) 22.0 (IBM-SPSS Inc., Chicago, IL, USA). In our behavioral analysis, differences in test–retest changes among the four groups were investigated, using one-way analysis of covariance (ANCOVA), with the differences between pre-test and post-test measures as dependent variables and pre-test scores as covariates (*P* < 0.05).

We have employed ANCOVAs instead of repeated measure ANOVAs to control the effects of pre-test scores in these kinds of intervention studies. Statistical experts strongly recommend to use ANCOVA instead of repeated measure ANOVA in this type of study design (Dimitrov and Rumrill 2003). With randomized designs, the purpose of ANCOVA is to reduce error variance (Dimitrov and Rumrill 2003). In a randomized controlled trial, pre-test scores should share no variance with the group. As a result of direct assignment, the expected mean values of pre-test scores in each group should be the same (plus or minus random error) (Miller and Chapman 2001). When ANCOVAs are used in this way, it serves as a legitimate and useful noise reduction technique for evaluating the relationship of the group differences and dependent values. It has been argued that even when group differences in covariates (in the case, pre-test scores) are substantive, if the group differences could not have caused the differences in covariates (pre-test scores), ANCOVA may be legitimate (Miller and Chapman 2001). Regardless, in this study, there were no significant differences in pre-test scores among the four groups (Table 1).

**Table 1:** The mean ± SD of the difficulty levels of the most difficult performances of all subjects (the highest difficulty level at which the subjects still achieved at least five correct answers to the 10 problems in each set of stimuli in the training tasks, between the first three and last three training sessions)

	First three sessions	Last three sessions
Active-control training (semitones (#) higher than the original pitch)	12.72 ± 2.49	19.36 ± 5.32
Training with TCSSL (× times faster than the original speed)	2.09 ± 0.38	2.97 ± 0.55
Reading-aloud training [time to finish reading the given text (s)]	619 ± 82	549 ± 71
Shadowing training (× times faster than the original speed)	0.92 ± 0.13	1.59 ± 0.30

There were numerous potential contrasts in this study because there were four groups with two factors (speeded listening and requirement of speaking). However, we only tested two contrasts to test our hypothesis. The first was the effect of the training with speaking (shadowing and reading aloud) versus training without speaking (TCSSL group, and active-control group), and the second was the shadowing-specific effect. In behavioral analyses, this effect was investigated using ANCOVA and by testing the interaction between the factor “presence of speaking” (shadowing, reading aloud, or other groups) and the factor “speeded listening (shadowing, TCSSL or other groups).” In the imaging analyses, this effect was investigated using the contrast [(shadowing – reading aloud) – (TCSSL – active control)] followed by possible post-hoc analyses. When significant effects were observed in these analyses, subsequent analyses were performed to see if there was a significant effect of shadowing training compared with the active-control group alone or a significant effect of reading-aloud training compared with the active-control group alone. The effects of TCSSL compared with the active control group (high-pitch training) were investigated by another study (the first author was T Maruyama who conducted procedures involving TCSSL) in the previous study (Maruyama et al. 2018) and there for the contrasts testing effects of TCSSL compared with the active control group (high-pitch training) were not investigated in this study. Rather, in the present study, the TCSSL group was used as one of the control groups for shadowing training as TCSSL training did not include simultaneous cognitive activities involving speaking.

When there were hypotheses, one-tailed tests were used as described previously (H. Takeuchi et al. 2011a; H. Takeuchi et al. 2011d; Klingberg et al. 2002; Klingberg et al. 2005). We hypothesized that training groups with speaking (the shadowing training and reading aloud training groups) would show greater improvement in their performance of working-memory tasks, English listening, and reading tests than in training groups without speaking. We additionally hypothesized that the training groups with speaking (shadowing training group and reading aloud training group) would show greater improvement in their performance of these tasks compared with the active control group.

In group-level imaging analyses, we tested for group-wise differences in changes in brain activity during n-back tasks and in rGMV across the entire brain.

In the group-level imaging analysis of rGMV, we tested for group-wise differences in the change in rGMV across the whole brain. We used the one-way ANOVA option in SPM8. In the imaging analysis, the effects of training were estimated by the same contrasts of behavioral analyses at each voxel. This analysis was limited to areas where the average rGMV value for the segmented and normalized mean images of pre-scans and post-scans of all participants was greater than 0.1. A multiple comparison correction was performed using the randomized (5000 permutations) nonparametric testing of the t score, using the distributed toolbox (http://dbm.neuro.uni-jena.de/tfce/). We applied a threshold of family-wise error corrected at *P* < 0.05. The standard cluster size test is inappropriate in voxel-based morphometry (Hayasaka et al. 2004), whereas a permutation-based test is assumed to be correct. The same principle was applied to our previous study, and the description of these methods were reproduced from this study (Maruyama et al. 2018). We used SPM12 for pre-processing of VBM and SPM8 for statistical analyses of the permutations because the first author was accustomed to writing script for statistical analyses. However, as long as the permutation test is used, the results will not be affected by the version of SPM.

In the analyses of functional activation, we performed voxel-wise ANCOVAs, using the changes in value of each measure from the pre-scans to the post-scans at each voxel as dependent variables, and the value for the pre-scan at each voxel as covariates. Under this paradigm, frame-wise displacements, reaction time, and accuracy of the corresponding task in the pre-scans, and changes of those measures from the pre-scans to the post-scans, were additionally added as covariates. Then, voxel-wise ANCOVAs were performed using Biological Parametrical Mapping (BPM) (Casanova et al. 2007) implemented in SPM8. For these analyses, regions with significance were inferred using cluster-level statistics (K. J. Friston et al. 1996a and b). Only clusters with *P* < 0.05 after correction for multiple comparisons at a cluster size with an uncorrected voxel-level cluster-determining threshold of *P* < 0.001 were considered statistically significant in this analysis. However, in the voxels of the areas that were regions with the a priori hypothesis (see Introduction), and in which significant group differences were found in VBM analyses, small volume correction was applied in the case of same group comparisons. Post-hoc analyses were performed to examine if functional plasticity occurred in areas where structural plasticity was observed. In other words, whole brain analyses and regions of interest analyses using small volume correction were conducted for the functional activation analyses of working memory (2-back) and for all the tested contrasts [speaking (+) v.s. speaking (−), shadowing vs. active control, reading aloud vs. active control]. Actual areas of masked regions of interest can be seen in Fig. 2. For these regions of interest, the *P* < 0.05 corrected for false discovery rate (FDR) was applied. In the analyses of functional activation, we used these statistical methods for corrections of multiple comparisons; this was because permutation-based corrections for multiple comparisons could not be used in BPM.

## Results

### Training data

During the 27-day intervention period, the mean number of sessions completed by the subjects in training was 25.97 ± 2.39; the value was 25.48 ± 2.79 in the active control training group; 25.39 ± 2.44 in the reading-aloud training group; and 25.31 ± 2.74 in the shadowing training group. Given that one subject could not complete more than 27 sessions, these results show the subjects were mostly able to perform the task properly. The level of performance (the highest difficulty level at which the subjects achieved at least five correct answers to the 10 problems in each set of auditory stimuli in the three training groups with auditory stimuli and the fastest speed with which the subjects could read aloud in the case of the reading-aloud training group) was significantly increased in the last three training sessions compared with the first three training sessions (paired *t*-test, *P* < 0.001, Table 1) in all 4 groups. The fact that subjects in the active-control group (which changed the pitch of the stimuli) faced this limitation suggests that when the pitch of the stimuli is increased, the subjects become unable to comprehend the stimuli. The descriptions of and the data from the TCSSL group and the active-control group in this subsection were reproduced from our previous study (Maruyama et al. 2018).

Furthermore, according to the questionnaire that was administered to the participants after the experiments, there were no significant differences among the four groups in their motivation toward the training tasks (*P* = 0.610, F = 0.609, one-way ANOVA), fatigue of the subjects after the training tasks (*P* = 0.753, F = 0.409), fatigue of the subjects after the training period (*P* = 0.699, F = 0.477), satisfaction with the training tasks (*P* = 0.190, F = 1.613), enjoyment during the training tasks (*P* = 0.551, F = 0.706), expectations of the effects of training on English reading skills (*P* = 0.387, F = 1.020), performance of the task of n-back working-memory task (*P* = 0.270, F = 1.324), performance of listening-span task (*P* = 0.129, F = 1.931), and performance of digit-span task (*P* = 0.817, F = 0.311). There was a difference in the expectation of effect of training on English listening (*P* = 0.017, F = 3.544) at the level of *p* < 0.05, uncorrected (the expectation in the reading-aloud group was smaller than in other groups).

### The effect of training with on psychological test performance

Among subjects who completed the study, those who misunderstood the rules of specific tasks or had missing data were excluded from individual analyses. In the TCSSL training group, 4 participants had invalid data in the n-back task, while none had invalid data in the listening span task, English listening span task, and English reading task. In the activate control group, 1 had invalid data in the n-back task, 4 in the listening span task, 1 in the English listening span task, and 2 in the English reading task. In the reading-aloud training group, 3 participants had invalid data in the n-back task, 2 in the listening span task, and none in the English listening span task and English reading task. Finally, in the shadowing training group, 2 participants had invalid data in the n-back task, 1 in the listening span task, and none in the English listening span task and English reading task. Data were valid in all other tasks among groups.

**Fig. 1:**
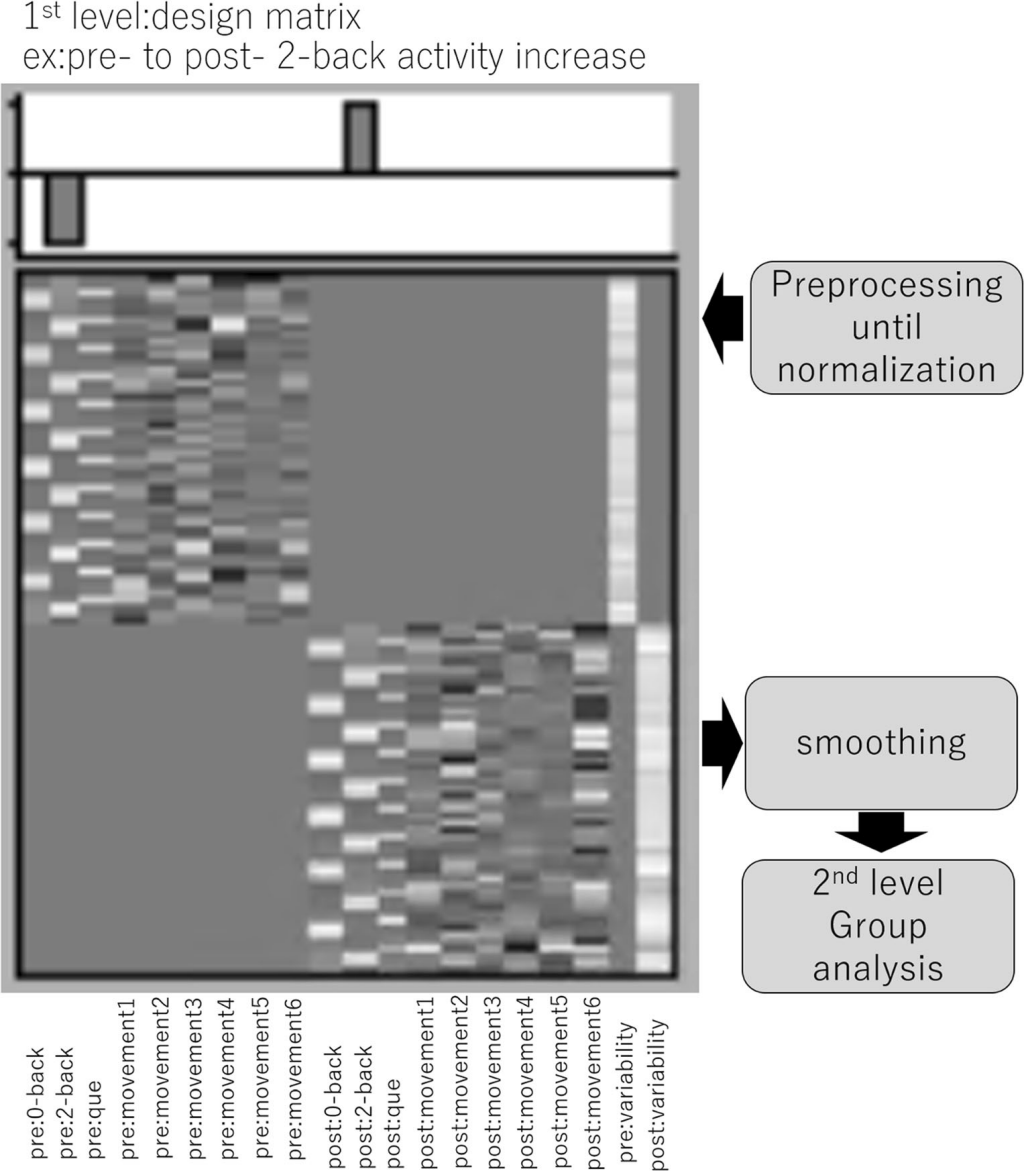
(a, b) Pre-training and post-training test scores of each training group. Bars show the mean values for each group. Error bars represent standard errors. Compared with the groups without the presence of speaking (TCSSL and active control), the groups with the presence of speaking (shadowing and reading aloud) showed tendency of greater test–retest differences in the digit-span scores (a) and significantly smaller test–retest differences in reaction time of the 2-back task (b)

In the behavioral analyses, we first investigated the main effect of speaking (shadowing and reading aloud versus TCSSL and active control). The results revealed that compared with the groups without speaking (TCSSL and active control), the groups trained with speaking (shadowing and reading aloud) showed a tendency for greater test–retest differences in the digit-span scores (Fig. 1a), and significantly smaller test–retest differences in the reaction time of the 2-back task (Fig. 1b). There were no other significant results or tendencies that were congruent with our hypothesis. These results could not be explained by preexisting differences (if any) between groups because the pre-test scores were controlled by ANCOVAs in these analyses. There were no preexisting group differences of these measures among the four groups (Table 2). For all statistical values obtained for group differences in pre-to-post changes of psychological scores, see Table 3. Accuracies of N-back tasks cannot be used as meaningful measures as they show apparent ceiling effects.

**Table 2:** Pre-test scores for behvioral measures (mean ± SD) of the subjects whose relevant data was obtained in both the pre-experiments and post-experiments

	Active control	TCSSL^b^	Reading aloud	Shadowing	Group differences(p value)^c^
0back – accuracy (%)	99.64 ± 1.57	99.93 ± 0.33	99.67 ± 1.05	99.75 ± 0.98	0.783
0back - RT	4486 ± 634	4708 ± 875	4820 ± 1316	4469 ± 722	0.451
2back - accuracy (%)	98.44 ± 4.48	98.75 ± 2.70	98.42 ± 2.84	99.54 ± 1.31	0.525
2back - RT	7183 ± 2137	7073 ± 1916	7792 ± 2673	6565 ± 1259	0.218
Digit span (score)	38.90 ± 7.49	37.17 ± 7.16	36.39 ± 6.09	36.38 ± 6.33	0.473
Listening-span test (score)	12.68 ± 3.34	12.79 ± 2.67	12.54 ± 2.91	12.00 ± 3.03	0.767
English listening test (score)	283.93 ± 47.25	268.1 ± 53.46	273.93 ± 54.96	276.72 ± 48.45	0.710
English reading test (score)	230.00 ± 60.29	232.93 ± 57.98	239.29 ± 59.02	233.28 ± 51.38	0.569
RAPM^a^ (score)	28.79 ± 4.80	26.61 ± 4.43	27.93 ± 3.43	28.14 ± 3.00	0.256
Word-Color task (items)	72.28 ± 6.21	67.69 ± 9.04	68.68 ± 8.66	69.48 ± 7.60	0.168
Color-Word task (items)	53.48 ± 5.65	52.97 ± 5.26	50.93 ± 6.96	53.17 ± 6.08	0.390
Reverse-Stroop task (items)	62.76 ± 5.58	60.90 ± 6.06	59.79 ± 10.31	60.55 ± 7.70	0.518
Stroop task (items)	51.76 ± 6.52	48.72 ± 6.75	47.07 ± 8.58	51.62 ± 5.63	0.013
Simple arithmetic (items)	32.52 ± 5.14	31.93 ± 5.15	31.21 ± 4.95	32.36 ± 5.18	0.784
Complex arithmetic (items)	6.72 ± 2.18	7.55 ± 4.45	6.98 ± 3.85	6.10 ± 2.27	0.439
Japanese reading comprehension (items)	13.66 ± 3.76	14.31 ± 4.42	13.25 ± 4.28	13.79 ± 4.54	0.832
SA creativity test (total grade)	25.34 ± 5.55	25.41 ± 6.12	26.18 ± 5.26	25.41 ± 6.65	0.947

^a^Raven’s Advanced Progressive Matrices

^b^Time-compressed speech training

^c^One-way ANOVA

**Table 3:** Change in scores of behavioral measures from the pre experiments to post experiments (mean ± SD)

	Active control	TCSSL^b^	Reading aloud	shadowing	hypothesis	Speaking vs.No speaking p value^c^	Interaction effect* P value^d^
0back - accuracy (%)	0.12 ± 1.66	−0.20 ± 0.86	0.20 ± 0.98	0.06 ± 1.15	-*	0.431	0.940
0back - RT	−16 ± 488	−36 ± 720	−367 ± 429	−135 ± 551	Two-tailed	0.051	0.376
2back - accuracy (%)	0.52 ± 3.03	−0.25 ± 3.4	1.25 ± 2.76	−0.54 ± 3.08	-*	0.425	0.584
2back - RT	−523 ± 1162	−585 ± 1329	−1324 ± 1288	−936 ± 1354	Reading (+) > Reading (−)	0.010	0.658
Digit span (score)	−0.14 ± 4.85	0.55 ± 6.46	1.68 ± 5.00	2.48 ± 5.66	Reading (+) > Reading (−)	0.074	0.785
Listening-span test (score)	0.28 ± 2.86	0.59 ± 3.33	0.46 ± 2.72	1.21 ± 2.93	Reading (+) > Reading (−)	0.540	0.785
English listening test (score)	−14.29 ± 33.24	−15.86 ± 61.55	−14.64 ± 30.24	−15.52 ± 37.42	Two-tailed	0.986	0.718
English reading test (score)	8.7 ± 43.28	−11.21 ± 56.12	0.71 ± 36.88	8.28 ± 29.43	Two-tailed	0.352	0.106
RAPM^a^ (score)	2.24 ± 2.60	2.55 ± 2.82	3.18 ± 2.84	2.31 ± 2.91	Two-tailed	0.395	0.580
Word-Color task (items)	4.90 ± 4.11	8.21 ± 9.04	5.64 ± 5.07	6.48 ± 4.49	Two-tailed	0.465	0.702
Color-Word task (items)	2.03 ± 3.79	0.86 ± 4.57	1.89 ± 4.22	2.07 ± 4.34	Two-tailed	0.576	0.337
Reverse-Stroop task (items)	2.59 ± 5.88	2.79 ± 4.44	2.61 ± 5.68	2.28 ± 5.69	Two-tailed	0.600	0.963
Stroop task (items)	1.93 ± 3.89	2.66 ± 3.98	3.29 ± 8.55	0.86 ± 4.03	Two-tailed	0.178	0.957
Simple arithmetic (items)	−0.09 ± 3.23	0.52 ± 2.96	0.73 ± 3.36	−0.09 ± 3.23	Two-tailed	0.444	0.883
Complex arithmetic (items)	0.81 ± 1.43	0.34 ± 1.34	0.91 ± 1.58	0.81 ± 1.43	Two-tailed	0.388	0.645
Japanese reading comprehension (items)	4.34 ± 3.50	4.07 ± 4.08	3.71 ± 4.37	3.52 ± 3.75	Two-tailed	0.408	0.961
SA creativity test (total grade)	0.00 ± 5.98	−1.10 ± 4.05	−1.21 ± 4.73	0.14 ± 3.66	Two-tailed	0.860	0.187

^a^Raven’s Advanced Progressive Matrices

^b^Time-compressed speech training

^c^Effects of the presence of speaking by one-way ANCOVA

^d^Effects of the interaction between the presence of speaking and speeded listening by one-way ANCOVA

^*^Accuracy showed ceiling effects, so the analyses are void

We next investigated the shadowing-specific effect as described in Methods. The results revealed no significant effects.

The post hoc analyses on the 2-back task and the digit-span task demonstrated that compared with the active-control group, the shadowing group showed tendency of greater test–retest differences in digit-span scores [*P* = 0.08uncorrected7 (uncorrected), *P* = 0.174 (Bonferroni corrected within these two post-hoc analyses of digit-span scores), F = 1.896], and that the reading-aloud group showed greater but statistically nonsignificant test–retest differences in digit-span scores (*P* = 0.169 (uncorrected), *P* = 0.338 (Bonferroni corrected within these two post-hoc analyses of digit-span scores), F = 0.934). In addition, compared with the active-control group, the shadowing group showed tendency of larger test–retest decrease in reaction time of the 2-back task (*P* = 0.088 (uncorrected), *P* = 0.176 (Bonferroni corrected within these two post-hoc analyses of 2-back task reaction time), F = 1.875), and the reading-aloud group showed a larger test–retest decrease of reaction time of the 2-back task that was statistically significant (*P* = 0.020 (uncorrected), *P* = 0.040 (Bonferroni corrected within these two post-hoc analyses of the 2-back task reaction time), F = 4.478).

### The effect of training on brain structure (regional gray matter volume)

**Fig. 2:**
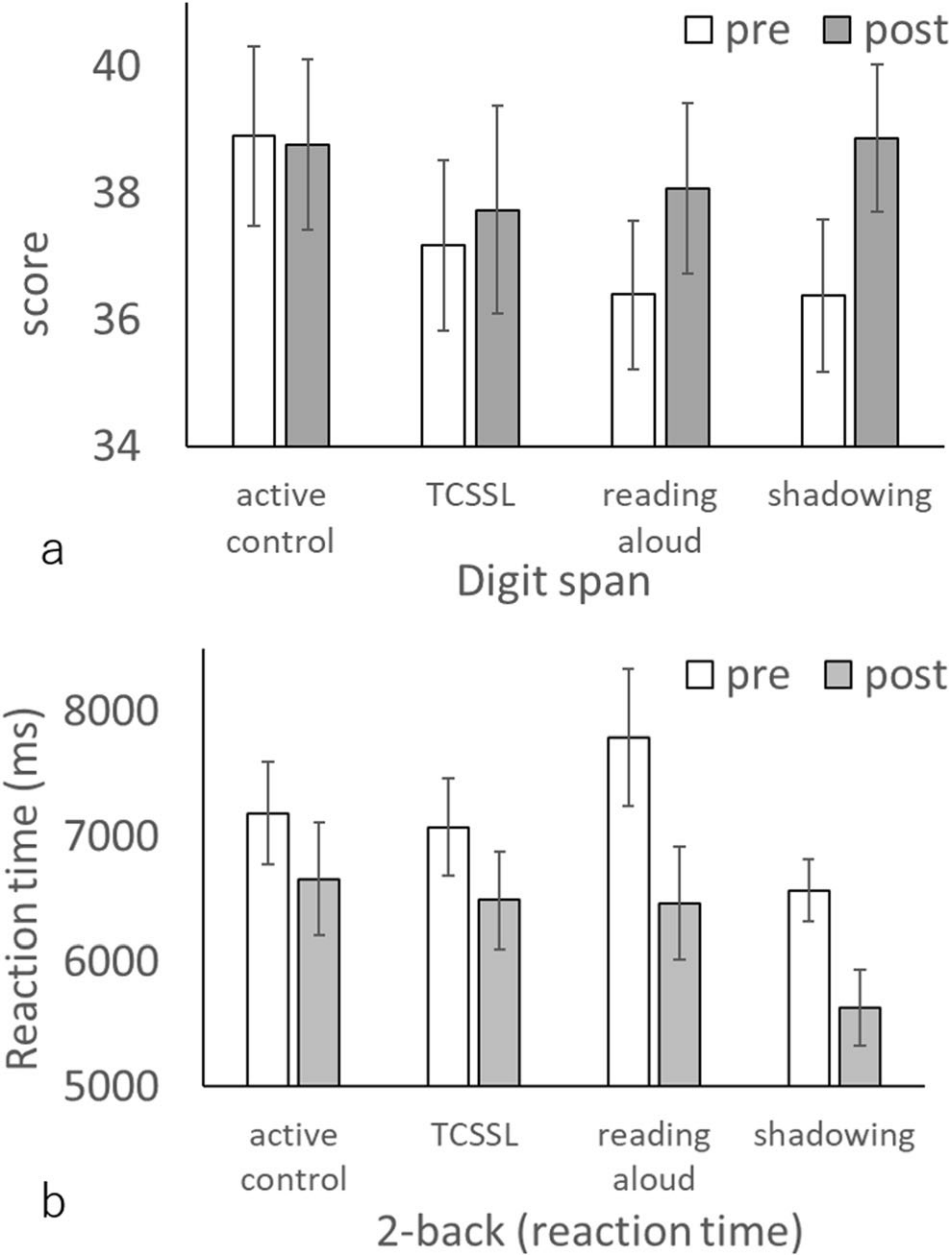
The effects of training on rGMV. The results shown were obtained using a threshold of *P* < 0.05, corrected for multiple comparisons based on 5000 permutations using T scores. (The upper panels) There was a greater decrease in rGMV in the groups with the presence of speaking compared with the groups without the presence of speaking (TCSSL, active-control group). This analysis was performed to identify differences in pre-training to post-training changes between the groups, as described in the Methods section. Training with the presence of speaking resulted in a decrease in rGMV in the areas around the area between the right insula and right superior temporal gyrus, as well as in the area within the left cerebellum. (The lower left figures) There was a greater decrease in rGMV in the groups that had training with shadowing compared with the active-control group. Training with shadowing resulted in a decrease in rGMV in the area within the left cerebellum. (The lower right figures) There was a greater decrease in rGMV in the groups that trained with reading aloud compared with the active-control group. Training with reading aloud resulted in a decrease in rGMV in the area between the right insula and right superior temporal gyrus

In the voxel-based morphometry analysis, we first investigated the main effect of the presence of speaking (shadowing and reading aloud versus TCSSL and active control) on brain structure (rGMV). The results showed that compared with the groups without the presence of speaking (TCSSL and the active-control groups), the groups with the presence of speaking (training of shadowing and the training of reading aloud) demonstrated a significantly larger training-related decrease in the rGMV in the area between the right anterior insula and the right superior temporal gyrus (Fig. 2; *x, y, z* = 36, 3, −16.5; t value = 4.40; *P* = 0.029, corrected for multiple comparisons using permutation of the t value (family-wise error), 37.125 mm^3^,with the threshold of *P* < 0.05, corrected for multiple comparisons) as well as in the area of the left cerebellum (Fig. 2; *x, y, z* = −24, −61.5, −22.5; t value = 4.26; *P* = 0.045, corrected for multiple comparisons, 20.25 mm^3^).

We next investigated the shadowing-specific effect [(shadowing – reading aloud) – (TCSSL – active control)]. The results did not reveal any significant effects.

Further, we investigated the effects of shadowing training in comparison to active control training. We found that compared with the active control group, the training of shadowing led to a significantly larger training-related decrease in rGMV in the area of the left cerebellum (Fig. 2; *x, y, z* = −25.5, −58.5, −22.5; t value = 4.98; *P* = 0.012, corrected for multiple comparisons, 2450.25 mm^3^). The peak voxel of this area corresponds to lobule VI of the left cerebellum.

Finally, we investigated the effect of training of reading aloud when compared with the active control training; we found a significantly larger training-related decrease in rGMV in the area between the right anterior insula and the right superior temporal gyrus (Fig. 2; *x, y, z* = 36, 1.5, −18; t value = 5.00; *P* = 0.009, corrected for multiple comparisons, 145.125 mm^3^).

### The effect of training on functional activity

**Fig. 3:**
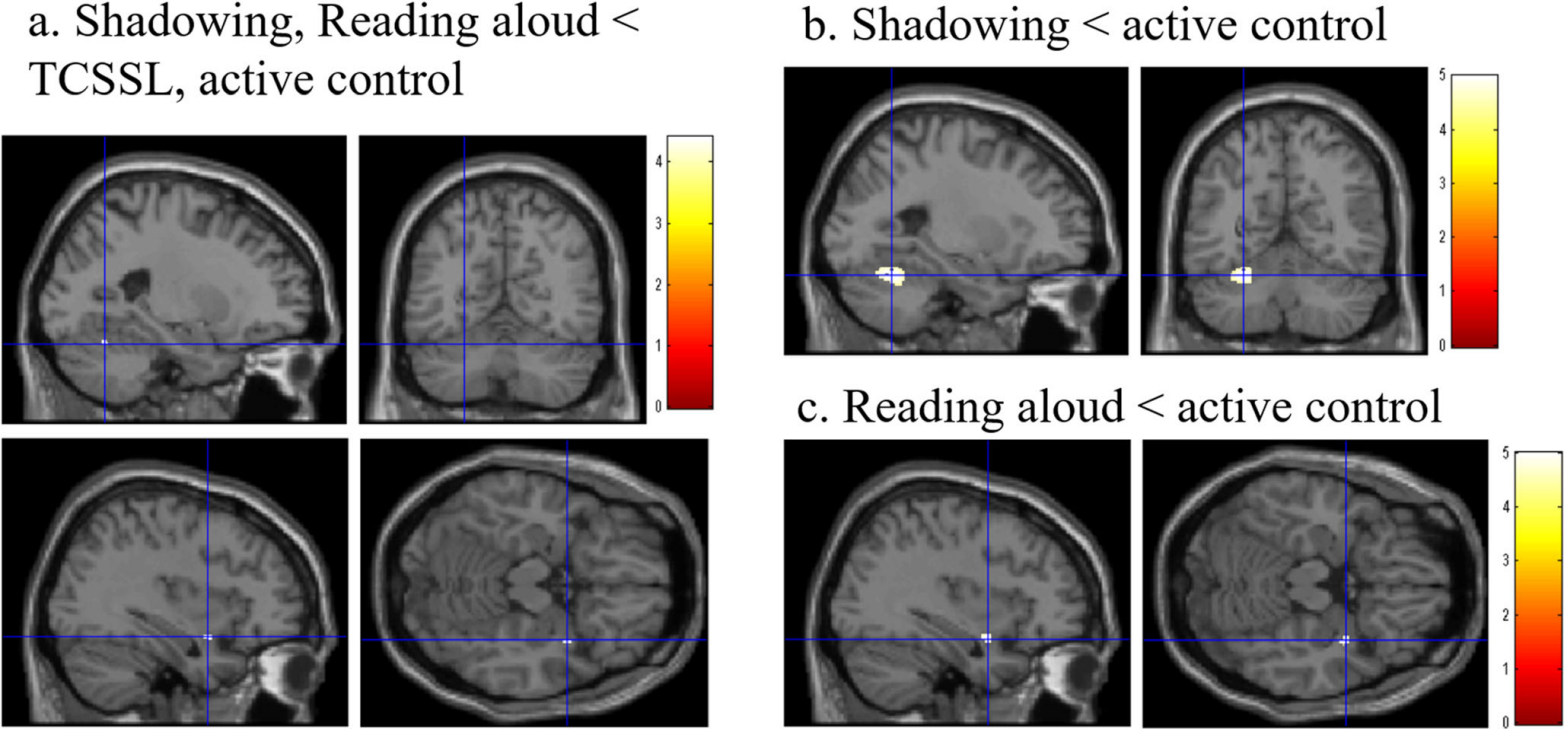
The effect of training on working-memory-related activity. The results shown were obtained using a threshold of uncorrected *P* < 0.05. (The left figures) There was a greater decrease in WM-related activity (activation during the 2-back task) in the group that received training with shadowing compared with the active-control group (this analysis was performed to identify differences in pre-training to post-training changes between groups, as described in the Methods section). Training with shadowing resulted in a decrease in WM-related activity in the area of the left cerebellum, where the effect of training on rGMV was found (P < 0.05, corrected for multiple comparisons within the areas of the significant effect). (The right figures) There was a greater decrease in WM-related activity (activation during the 2-back task) in the training with reading-aloud group compared with the active-control group. Training with reading aloud resulted in a decrease in WM-related activity in the area between the right anterior insula and right superior temporal gyrus, where the effect of training in rGMV was found (P < 0.05, corrected for multiple comparisons within the areas of the significant effect)

In the analyses of WM-related brain activity (brain activity during the 2-back task), in the whole brain analysis, there were no significant effects of the presence of speaking (shadowing and reading aloud versus TCSSL and active control), shadowing-specific effect [(shadowing – reading aloud) – (TCSSL – active control)], the effect of shadowing (shadowing versus active control), and the effects of reading aloud (reading aloud versus active control). The regions of interest analyses of functional activity within the areas of significant effects of the presence of speaking on rGMV (right anterior insula and the left cerebellum) demonstrated that there were no significant effects of the presence of speaking (shadowing and reading aloud versus TCSSL and active control) or a shadowing-specific effect [(shadowing – reading aloud) – (TCSSL – active control)]. However, the regions of interest analysis revealed there was a significant effect of shadowing training (shadowing versus active control) on brain activity in the left cerebellum (where the effect of shadowing training on rGMV was observed) [Fig. 3; *x, y, z* = −24, −63, −21; *t* = 1.78; *P* = 0.041, corrected for multiple comparisons (FDR), 27 mm^3^], as well as in the right anterior insula (where the effect of reading aloud training on rGMV was observed) [*x, y, z* = 36, 3, −18; *t* = 2.03; *P* = 0.041, corrected for multiple comparisons (FDR), 27 mm^3^]. Further, analysis on regions of interest revealed a significant effect of reading-aloud training (reading aloud versus active control) on brain activity of the area between the right anterior insula and right superior temporal gyrus [*x, y, z* = 36, 3, −18,; *t* = 2.43; *P* = 0.019, corrected for multiple comparisons (FDR), 27 mm^3^].

In the analyses of brain activity (using whole brain analysis) of simple cognitive processing (brain activity during the 0-back, non-WM task), there were no significant effects of the presence of speaking (shadowing and reading aloud versus TCSSL and active control), shadowing-specific effect [(shadowing – reading aloud) – (TCSSL – active control)], the effect of shadowing (shadowing versus active control), and the effects of reading aloud (reading aloud versus active control).

## Discussion

The present study newly investigated the effects of intensive adaptive training of shadowing of second language and training of reading aloud of second language on working-memory capacity, brain structures, and neural systems related to working memory. The results were partly congruent with our hypothesis. The results showed that, compared with the groups without the presence of speaking (TCSSL and active control), the groups with the presence of speaking (shadowing and reading aloud) showed tendency of greater increase in working-memory performance (digit-span scores) and significantly greater increase in another working-memory performance, reduction of the reaction time of 2-back task. These changes cannot be explained by preexisting group differences because this is a randomized controlled study. These significant changes in psychological metrics are consistent with our hypothesis, although the differences did not reach significance in the case of digit span score. Given our hypothesis, we expect the results may reach significance if the sample size is increased. On the other hand, the lack of significance or tendency in the listening span task is contrary to our hypothesis. Imaging analyses revealed that compared with the active-control group, the shadowing group showed both a decrease in rGMV (gray matter structure) and in brain activity during the WM task (2-back task) in the left cerebellum, which is a part of the phonological loop. In addition, we found that compared with the active-control group, the reading-aloud group both showed decreases in rGMV and brain activity during the WM task (2-back) task in the area between the right anterior insula and right superior temporal gyrus, which are parts of the perisylvian areas of the phonological loop. These results are in accord with our hypothesis, which predicted neural plasticity of functional activation and gray matter structural changes in neural systems of the phonological loop (perisylvian areas and the cerebellum) following training with the presence of speaking (reading aloud and shadowing).

Changes in rGMV and WM-related brain activity that were related to shadowing were observed in the area of the left cerebellum, which plays an important role in the articulatory rehearsal system of the working-memory system. The model of working memory involves a central executive system and sub-storage systems, and the phonological loop is one such sub-storage system (A. D. Baddeley 2017). In the model of working memory, the phonological loop comprises of a phonological store, which can hold memory traces for a few seconds before they fade, and an articulatory rehearsal process that is analogous to subvocal speech (A. Baddeley 2003, 2012). The cerebellum is assumed to play a key role in this articulatory rehearsal process (A. Baddeley 2003; Alderson-Day and Fernyhough 2015; Marvel and Desmond 2010). It is possible that shadowing training, which is assumed to engage this system, might lead to the use-dependent neural plasticity of this area and to increased neural efficiency that subsequently result in decreased activity during the task (Neubauer and Fink 2009). The peak voxel of significant findings on the cerebellum (*x, y, z* = −25.5, −58.5, −22.5) corresponds to lobule VI of the left cerebellum. A previous study utilizing the data of several hundred subjects’ data (Guell et al. 2018) and meta-analyses (Keren-Happuch et al. 2014; Stoodley and Schmahmann 2009) revealed the associations between each subregion of the cerebellum and functional activations. These studies showed that the areas of lobule VI close to this voxel showed activation in response to motor action, particularly tongue movement, language, and working memory. However, this is a reverse inference; the cerebellum is involved in a wide range of functions other than the articulatory rehearsal system; thus, other mechanisms are possible.

Changes in rGMV and WM-related brain activity, which has been associated with shadowing, were observed in the area between the right anterior insula and the right superior temporal gyrus, which plays an important role in the phonological loop of the working-memory system. (Schulze et al. 2011). The right anterior insula is suggested to be involved in the intonation contours of verbal utterances and musical melodies (Ackermann and Riecker 2004; Chang and Kuo 2016), and it is considered to lead the process of word generation (Indefrey and Levelt 2000; Oh et al. 2014). It is also hypothesized that the right anterior insula/medial frontal operculum provides linkage across systems, supporting task demands and attention systems during word recognition. Thus, this area can be regarded as one of the articulatory rehearsal systems. However, it has been shown that the right anterior insula/medial frontal operculum are functionally connected with other frontal regions implicated in executive function, and not just with brain regions responsive to stimulus salience. Furthermore, this area exhibits significantly correlated activity with other brain regions specifically engaged during tasks with varying perceptual and behavioral demands (Eckert et al. 2009). Therefore, the involvement of this area with other functions is also possible. Perhaps, shadowing training, which was assumed to engage this system, might have led to the use-dependent neural plasticity of this area and led to increased neural efficiency, which led to decreased activity during the task (Neubauer and Fink 2009).

The present results demonstrated that training with speaking (shadowing and reading aloud) both led to a decrease in rGMV of the relevant areas, similar to what was observed for the training of TCSSL in our previous study, but the reason for this change remains unclear. As described in our previous study (Maruyama et al. 2018), we speculated that the usage-dependent adaptive selective elimination of synapses (P. R. Huttenlocher and Dabholkar 1997; Bastrikova et al. 2008), which sculpt neural circuitry (Hensch 2004; Peter R Huttenlocher 2013), underlies the training-mediated decreases in rGMV. Consistent with this idea, a decrease in rGMV after cognitive training or other interventions that have either been shown or were assumed to be associated with preferable cognitive changes or learning, have been occasionally observed (H. Takeuchi et al. 2011a; H. Takeuchi et al. 2011d; Quallo et al. 2009; May et al. 2007). However, other studies reported that mild mid-term training mainly led to an increase in rGMV (Ilg et al. 2008; Driemeyer et al. 2008; Boyke et al. 2008; H. Takeuchi et al. 2013; Hikaru Takeuchi et al. 2014). On the basis of observations from a number of studies, we previously proposed that these differences could be explained by nonlinear changes in rGMV (an initial increase followed by a decrease), which were affected by the length and intensity of training (H. Takeuchi et al. 2011d). Consistently, it has been shown that learning new processes can lead to a transient increase in spine formation and that this rapid spinogenesis is followed by enhanced spine elimination (Xu et al. 2009). Further, in this case, the elimination of spines during subsequent training has largely been restricted to spines that existed before training (Xu et al. 2009). It is possible that second-language training by those well-experienced in the second language largely employed the spines that existed before training, which may have led to a phase where elimination became dominant. These theories are, however, purely speculative and should be verified in future animal studies.

This study had a few limitations, which have been addressed below and also in our previous study (some descriptions in this paragraph were reproduced from our previous study). First, this study did not include a passive control group due to the practical limitation of research resources; we thus set the active-control training group. Although the active-control training was designed to be ineffective, it has not been established whether this was truly the case. However, the effect on working memory in the high pitch active control training group may be unlikely. This is due to the fact that in our previous study, which utilized a very much similar trial design (2 outcome measures were divided by 4-week intervals), the passive control group improved by a score of 1.1 in the computerized digit-span performance (H. Takeuchi et al. 2013) as opposed to improvement of −0.1 score in the high pitch active control training group in the present study. Furthermore, we could not control for the amount of English the subjects in each group received in this experiment. This type of intervention study requires many resources, and it is difficult to create control groups for all confounding factors. In our experimental design, we attempted to control the training time as much as possible (though the training time should depend on the eagerness and skills of the subjects), but the amount of exposed English stimuli could not be controlled (the active-control group received the least stimuli, whereas the TCSSL group received the most stimuli). However, we believe that like other factors that we could not control (such as the pitch of the stimuli), the amount of English stimuli was not as important in this study because only subjects with relatively high levels of English skills and who studied the language for at least 6 years were eligible to participate in this study. Therefore, it would not be likely that receiving normal English auditory stimuli that were easy enough to comprehend at a normal speed for a short period of time, would substantially affect neural mechanisms. However, other training methods for higher order cognitive functions have been shown to improve cognitive functions even after training for 1–5 weeks (H. Takeuchi et al. 2011d; Jaeggi et al. 2011). In addition, meta-analysis showed only small associations of training sessions with effect sizes for far transfer effects among the conducted working memory studies (Au et al. 2014). We, along with other researchers, used representative groups of working memory training utilizing the 3–5 week training paradigms (Jaeggi et al. 2011; Klingberg et al. 2002; Westerberg et al. 2004; H. Takeuchi et al. 2013). Thus, 4-week training was considered sufficient to detect the far transfer effects of unexperienced cognitive training toward working memory measures. In terms of sample size, larger sample sizes tend to increase the statistical strength of true positive results. The statistical significance in this study was marginal. Additionally, the effect sizes of far transfer effects of cognitive training in normal young individuals are limited even in cases of established training regimes (Au et al. 2014), hence; larger sample sizes are desired. In this study, we recruited 119 participants. Enormous amounts of human resources (due to the instruction and measurement procedures) and economic resources (mainly monetary rewards for participations of 1-month intervention studies) were required for each participant. As such, it is difficult to find working memory training studies, especially using MRI, with this sample size. Due to multiple control groups, dividing the effects of fast listening and fast reading led the creation of four groups. Recruiting more individuals to increase the sample size was not possible for this interventional study. Future studies or meta-analyses may need to utilize a larger bulk of data.

In summary, the present study investigated the effects of training involving shadowing and reading aloud of text in a second language on language skills, working memory, and neural mechanisms in young adults. Partly consistent with our hypotheses, these training methods showed main effects on some working-memory performances. They did not show effects on second-language skills. Shadowing training led to the change of brain structure (rGMV) and neural activity related to working memory in the cerebellum, which play an important role in the phonological loop. Reading-aloud training led to the change of rGMV and neural activity related to working memory in the right insula, which also play an important role in the phonological loop. The observed neural changes caused by these training methods may offer new insights into how these training could lead to working-memory performance changes.

## Electronic supplementary material

ESM 1 (DOCX 28 kb)
