# Supplementary material for: Effects of training of shadowing and reading aloud of second language on working memory and neural systems
Source: Brain Imaging Behav. 2020 Jul 23;15(3):1253–69. doi: 10.1007/s11682-020-00324-4 (PMC8286220; doi:10.1007/s11682-020-00324-4)
Supplement: Supplementary file 1 — (DOCX 28 kb) [file 11682_2020_324_MOESM1_ESM.docx]

**Supplementary online material**

**Supplemental methods**

**English stimuli of training tasks.**

Some auditory English stimuli files (for the shadowing, TCSSL, and active control) and English texts (for the reading aloud) from Voice of America Special English (http://learningenglish.voanews.com/), Nihon Housou Kyokai (NHK; Japan Broadcasting Cooperation) news (Torikai and Ito, 2013), and NPR (<http://www.npr.org/>) were made available to the subjects on each training day (the details are described below). The English stimuli files from Voice of America Special English were used by the subjects with a beginner level of English, those from NHK news by the subjects with an intermediate level of English, and those from National Public Radio news by the subjects with a high level of English. Levels of English comprehension were determined through preliminary investigations and classified according to the TOEIC listening test (described later), with a score of 0–300 indicating a beginner level, 300–400 as an intermediate level, and 400–495 as a high level. A score was assigned simply to supply an appropriate level of difficulty at a normal speed. If the text was too difficult, then subjects were not able to comprehend the stimuli at a normal speed. If the text was too easy, then the task was not sufficiently challenging even when speeded up (within the limitation of the software and experimental procedure). It was not feasible to recruit subjects with approximately equivalent levels of English comprehension and within the same age range willing to participate in a one-month intervention experiment. Therefore, we could not use uniform English material for all subjects. On day 1, the subjects undertook the TOEIC practice exam (Nakamura et al. 2008) and received auditory English stimuli files (for all groups except the reading aloud group) or English texts (for the reading-aloud group) for training based on the score of this test. Each English stimuli file took approximately 10 min to complete. Everyday, the TCSSL training group was given six stimuli (auditory files), the shadowing and active-control group was given four (auditory files), and the reading-aloud group was given three (written texts). Different number of files were given because of time constraints (as it took less time to complete each training session in the TCSSL training group because of faster stimulus presentation and more time to complete each training session in the reading training group). Subjects were instructed to listen to the English stimuli files on their PC or tablets and perform the instructed tasks.

The same English stimuli and Japanese answers were used for all training tasks (Auditory files for the training group of shadowing, TCSSL, control (pitch), and written texts for reading). As described above, however, the number of stimulus files differed among groups. The English-language files used for training groups requiring more stimuli (such as the shadowing group) included all those used for training groups requiring fewer stimuli (such as reading group) plus additional English files. Each stimulus file consists of multiple news items, each consisting of a continuous series of sentences.

**Details of training tasks.**

(a) The descriptions in this subsection were reproduced from our previous study (Maruyama et al. 2018). We chose 10 English target sentences (such as “Smartphones are ruining the concert-going experience”) from each English auditory file and translated each into Japanese. The subjects were given the text files comprising 10 of these translated Japanese target sentences and assigned question numbers 1–10, for which the subjects were instructed to identify the English target sentences that corresponded to the given 10 Japanese target sentences while they listened to the auditory file and then asked to record their voice saying “Yes” when they found the given Japanese target sentence. If the subjects were unable to identify the given target sentence, they were asked to ignore the target sentence and proceed to the next given target sentence. In the English auditory file, the English target sentences that corresponded to the given Japanese target sentences appeared in numerical order (in other words, target sentence “1,” target sentence “2,” …, and target sentence “10” appeared in this order in each English file). For the subjects to judge when the given 10 Japanese target sentences appeared, 8 s after the English target sentences that corresponded to the given Japanese target sentences streamed, the target sentence number streamed (“1,” “2,” and “3” and so on). In other words, 8 s after target sentence “1” streamed, the spoken word “one” (in Japanese) streamed. If the subjects could record their voice saying “Yes” before the corresponding question number streamed, this established that they could identify the given Japanese target sentence that corresponded to the question number and had found it correct. If the subject stated the word “Yes” at an inappropriate time, the response was regarded as incorrect and that the subject was unable to identify the correct answer. Subjects were required to mark their own answers and send the scores and the recorded files to the experimenters. There were 10 target sentences per English file, and each day the subjects in the training with TCSSL group listened to six English files and were instructed to complete the associated tasks.

The stimulus speed was modulated according to how well the subjects could detect the given text target sentences using the free sound software Windows Media Player (Microsoft Corporation, Redmond, WA, USA). For the first trial on the first training day, the subjects were instructed to listen to the auditory English stimuli files at 1.5 times faster than the original speed. Subsequently, the stimulus speed was modulated on the basis of the performance in the previous trial. If the subject’s performance (the number of the target sentences that the subjects identified correctly) in the previous trial is expressed as X (which has a value between 0 and 10 as there were only 10 target sentences in each trial) and the listening speed for that trial was Y_t_ (i.e., in the trial, the speed of the stimuli was Y_t_ times faster than the original speed), then the speed of the task in the subsequent trial (Y_t+1_) became [Y + 0.05 × (X − 5)], faster than the original speed. When the subjects’ performance was graded as a little bit good, the difficulty of the task was increased a little bit. Through this procedure, the speed was modulated finely. But when the task was too easy or too difficult, the difficulty of the task was rapidly adjusted to an appropriate level. This procedure was used in our previous studies (Hikaru Takeuchi et al. 2014) and was found to effectively adjust the difficulty of the task. When the speed of the task became twice as fast as the original speed, subjects were instructed to modulate the speed of the stimuli, which were twice as fast compared to the original speed.

(b) In the shadowing training group, 4 auditory stimuli files were given to subjects each day. the confirmation tasks were similar to the training tasks of TCSSL. However, in the shadowing group, in addition to the procedure of the training tasks of TCSSL, subjects were asked to perform shadowing (reading aloud the target sentences they heard from the auditory English stimuli).

Furthermore, subjects were asked to subjectively rate how much they could perform shadowing (through 100%–0%). For example, if the subjects rated that they could perform shadowing for 70% of the stimuli, then the performance level was 7, and if the subjects rated they could perform shadowing for 30% of the stimuli, then the performance level was 3. For the task that was common to the TCSSL training task, the performance level corresponded to the number of the target sentences that the subjects identified correctly. In the shadowing training, the difficulty level of the stimuli of the next trial was determined by the lower performance level of the two tasks (shadowing and target sentence finding). If the subject’s performance (lower performance level of the two tasks) in the previous trial is expressed as X (which has a value between 0 and 10) and the listening speed for that trial was Y_t_ (i.e., in the trial, the speed of the stimuli was Y_t_ times faster than the original speed), then the speed of the task in the subsequent trial (Y_t+1_) became [Y + 0.025 × (X − 5)], faster than the original speed. Additionally, in shadowing training, for the first trial on the first training day, the subjects were instructed to listen to the auditory English stimuli files at 0.8 times faster than the original speed. When necessary, the experimenter gave feedback on whether the subjective evaluation by the subjects on their own performance level of shadowing was accurate.

(d) In the control group, the pitch was modulated according to the performance of the task such that subjects had to listen to the English auditory files as high in pitch as possible while maintaining their task performance. 3 auditory files were given to subjects each day. The descriptions in this subsection were reproduced from our previous study (Maruyama et al. 2018). The English auditory files were streamed, and the pitch was modulated using the free sound software Hayaemon (<http://soft.edolfzoku.com/hayaemon2/>). At first, the subjects were instructed to listen to auditory English stimuli files at 10 semitones (10#) higher than the original sound. One unit for modulation of the task difficulty was 0.5 semitone (#), and the pitch of the task was decreased or increased in a similar manner to the modulation of speed used for the training with TCSSL group, with the stimuli pitch modulated on the basis of how well the subjects could detect the given text target sentences. Specifically, if the subjects’ performance in the previous trial was X (a value between 0 and 10, as there were only 10 target sentences in each trial) and the current listening pitch was Y (e.g., if Y = 12, subjects had to listen to auditory English stimuli files at 12 semitones (10#) higher than the original sound), then the pitch in the subsequent trial became [Y + 0.5 × (X − 5)].

**Details of the N-back task (fMRI task)**

The n-back task was performed during an fMRI scanning, as described in our previous study (Takeuchi et al. 2011b, 2011a; H. Takeuchi et al. 2014). We have provided the same information here for the convenience of the readers. Participants received instructions and practiced the tasks before entering the scanner. During scanning, they viewed stimuli on a screen via a mirror mounted on a head coil. Visual stimuli were presented using Presentation software (Neurobehavioral Systems, Inc., Albany, CA, USA). A fiber-optic, light-sensitive key press interface with a button box was used to record participants’ behavior.

We used a simple block design and the n-back WM task (Callicott et al. 1999) to tap brain activities during the WM task. There were two conditions (0- and 2-back). Each condition had six blocks, and all n-back tasks were performed in one session. The subjects were instructed to recall stimuli [visually presented four types of Japanese letters of vowels) seen “n” times previously. In the 0-back task, subjects were instructed to determine if each presented letter was one of the target stimuli. Two buttons were used during the 0-back task, and the subjects were asked to push the first button when the target stimuli were presented and the second button when the other stimuli were presented. In the 2-back task subjects were instructed to determine whether each presented letter was the same as that presented two stimuli previously and to push the second button when the currently presented stimuli and the stimuli presented two letters previously were different. Our version of the n-back task was designed to require individuals to push buttons continuously during the task period. The task level of the memory load was shown in the upper part of the screen 2 s before the task started (this 2 sec period was the cue phase) and remained at the same place on the screen during the task period. Each letter was presented for 0.5 s, and a fixation cross was presented for 1.5 s between each item. Each block consisted of 10 stimuli. Thus, each block lasted for 20 s. A baseline fixation cross was presented for 13 s between the task and the presentation of the next condition’s task level (2 s). Thus, the rest period lasted for 15 s. There were six blocks for each 2- and 0-back condition.

In both of conditions, subjects were instructed to push the appropriate buttons as fast as possible.

**Rationale for use of DTI images in the normalization procedure for BOLD images.**

As described in our previous study (Takeuchi et al. 2011c), we did not use T1-weighted structural images for the process of coregistering because visual inspections suggest the process of coregistering the BOLD images taken in our studies to the T1-weighted structural images used in our laboratory often fails. This failure could be attributable to differences in the two images caused by susceptibility-induced signal losses and geometric distortions that occur more strongly in EPI using high-Tesla MRI (Liu and Ogawa 2006). Since similar distortion problems exist in DTI images, the coregistration of BOLD images toward DTI images did not cause such problems.

**References**

Callicott, J. H., Mattay, V. S., Bertolino, A., Finn, K., Coppola, R., Frank, J. A., et al. (1999). Physiological characteristics of capacity constraints in working memory as revealed by functional MRI. *Cerebral Cortex, 9*(1), 20-26.

Liu, G., & Ogawa, S. (2006). EPI image reconstruction with correction of distortion and signal losses. *Journal of Magnetic Resonance Imaging, 24*(3), 683-689.

Maruyama, T., Takeuchi, H., Taki, Y., Motoki, K., Jeong, H., Kotozaki, Y., et al. (2018). Effects of time-compressed speech training on multiple functional and structural neural mechanisms involving the left superior temporal gyrus. *Neural Plasticity, 2018*, Article ID 6574178, 6574112 pages.

Nakamura, S., Anderton, S., Kanzaki, M., & Kobayashi, M. (2008). TOEIC(R) test new best triple practice exam. Japan Times.

Takeuchi, H., Taki, Y., Hashizume, H., Sassa, Y., Nagase, T., Nouchi, R., et al. (2011a). Effects of training of processing speed on neural systems. *Journal of Neuroscience, 31*(34), 12139-12148.

Takeuchi, H., Taki, Y., Hashizume, H., Sassa, Y., Nagase, T., Nouchi, R., et al. (2011b). Failing to deactivate: the association between brain activity during a working memory task and creativity. *Neuroimage, 55*(2), 681-687.

Takeuchi, H., Taki, Y., Hashizume, H., Sassa, Y., Nagase, T., Nouchi, R., et al. (2011c). Failing to deactivate: the association between brain activity during a working memory task and creativity. *Neuroimage, 55*(2), 681-687.

Takeuchi, H., Taki, Y., Nouchi, R., Hashizume, H., Sassa, Y., Sekiguchi, A., et al. (2014). Associations among imaging measures (2): The association between gray matter concentration and task-induced activation changes. *Human Brain Mapping, 35*(1), 185-198.

Takeuchi, H., Taki, Y., Nouchi, R., Hashizume, H., Sekiguchi, A., Kotozaki, Y., et al. (2014). Effects of Multitasking-Training on Gray Matter Structure and Resting State Neural Mechanisms. *Human Brain Mapping, 35*(8), 3646-3660, doi:10.1002/hbm.22427.
